# Torque-induced precession of bacterial flagella

**DOI:** 10.1038/srep18488

**Published:** 2015-12-22

**Authors:** Yuji Shimogonya, Yoichiro Sawano, Hiromichi Wakebe, Yuichi Inoue, Akihiko Ishijima, Takuji Ishikawa

**Affiliations:** 1 Frontier Research Institute for Interdisciplinary Sciences, Tohoku University, Sendai 980-8578, Japan; 2 Graduate School of Life Sciences, Tohoku University, Sendai 980-8577, Japan; 3 Institute of Multidisciplinary Research for Advanced Materials, Tohoku University, Sendai 980-8577, Japan; 4 Graduate School of Frontier Biosciences, Osaka University, Osaka 565-0871, Japan; 5 Department of Bioengineering and Robotics, Tohoku University, Sendai 980-8579, Japan

## Abstract

The bacterial flagellar motor is an ion-driven rotary machine in the cell envelope of bacteria. Using a gold nanoparticle as a probe, we observed the precession of flagella during rotation. Since the mechanism of flagella precession was unknown, we investigated it using a combination of full simulations, theory, and experiments. The results show that the mechanism can be well explained by fluid mechanics. The validity of our theory was confirmed by our full simulation, which was utilized to predict both the filament tilt angle and motor torque from experimental flagellar precession data. The knowledge obtained is important in understanding mechanical properties of the bacterial motor and hook.

The flagellar motor of swimming bacteria, such as *Escherichia coli* (*E*. *coli*) and *Serratia marcescens (S. marcescens)*, is a rotary molecular machine that is powered by an ion flux; the diameter of the motor is ~45 nm. The torque generated by the motor is transmitted to a helical flagellar filament through a hook, which allows the filament to rotate as a screw propeller so that the bacteria can swim in fluid^1^. Due to its biological importance as well as engineering interest, the motor characteristics have been investigated experimentally for various types of cells, using the stuck cell and bead assay techniques^2^,^3^,^4^,^5^. The fluid mechanical aspects of flagellar rotation have also been examined^6^,^7^,^8^,^9^,^10^,^11^.

When we performed a bead assay, in which the cell body was affixed to a glass surface to observe the rotation of a truncated flagellum via the positioning of a 250 nm-diameter gold nanoparticle, we often observed that the filament motion consisted of two types of rotation: *spin* and *revolution*, which resulted in precession, as shown in Videos S1–S3 and Fig. S1 in Supplementary Information. Since rotational radius over 200 nm in the precession is much larger than the single-mode rotation in the previous analysis of torque-speed curves of Inoue *et al.*^5^, the mechanism of the precession has not been explored by the experimental study. In the theoretical work, Vogel and Stark^12^ described briefly the precession in torque-driven filament motion; however, questions remain regarding the motion mechanism. A physical picture of the precession motion must be established to fully understand flagellar motor characteristics, flagellar hook rigidity, and the interactions between flagella.

In this paper, we discuss the physics of torque-induced precession of a bacterial flagellum by combining full simulations, theory, and experiments. The results of the full simulation showed that the mechanism of precession could be well explained by fluid mechanics. The relationship between spin and revolution movements was clarified in detail; our simple theory also well described the basic characteristics. Finally, we applied our theory to predict the tilt angle of filaments and the motor torque of chimeric motors expressed in *E. coli*., to demonstrate the usefulness of our theory.

## Results and Discussion

**Figure 1:**
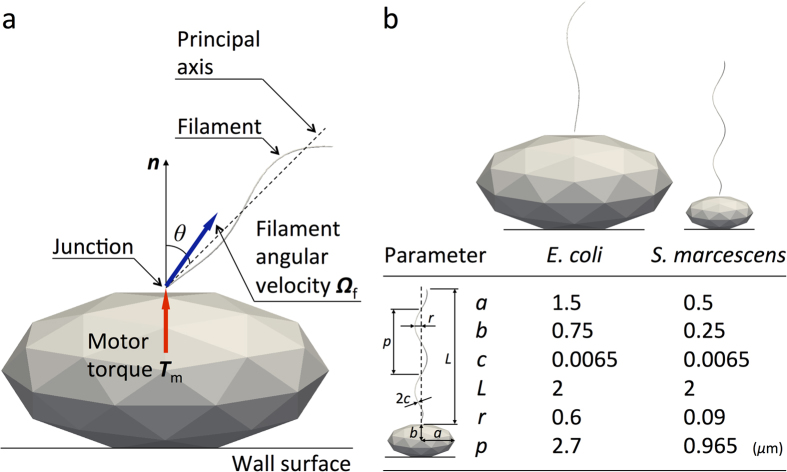
Bacterial model. (**a**) Schematic diagram of the problem setting. (**b**) Geometric parameters of *E*. *coli*^18^,^20^ and *S. marcescens*^19^,^20^ models, where *a* and *b* are polar radius and equatorial radius of a prolate spheroidal cell body, respectively, *c* is radius of a filament, *L* is helix length, *r* is helix maximum radius, and *p* is pitch.

We assumed that the cell body was stuck to a wall surface, as shown in Fig. 1a, similar to the set-up used in previous experimental studies^2^,^3^,^4^,^5^; this allowed for a better comparison between the numerical and experimental results. To further simplify the problem, we calculated the motion of a single flagellum; the interactions among multiple flagella were neglected. The cell body and flagellar filament were assumed to be rigid. The flagellar filament and motor were connected by a hook, which was modeled as a universal joint^13^ without bending rigidity. A constant torque vector ***T***_m_, generated at the junction by the motor, was applied in the normal direction ***n*** with respect to the cell surface, as shown in Fig. 1a. In this configuration, the flagellar filament exhibits torque-induced precession with angular velocity ***Ω***_f_ and tilt angle *θ*, the angle between the principal axis of the filament and the normal axis. We should note that the principal axis of the filament and the angular velocity axis do not necessarily orient in the same direction. Figure 1b shows the *E*. *coli* and *S. marcescens* bacterial models used in this study (see Materials and Methods).

**Figure 2:**
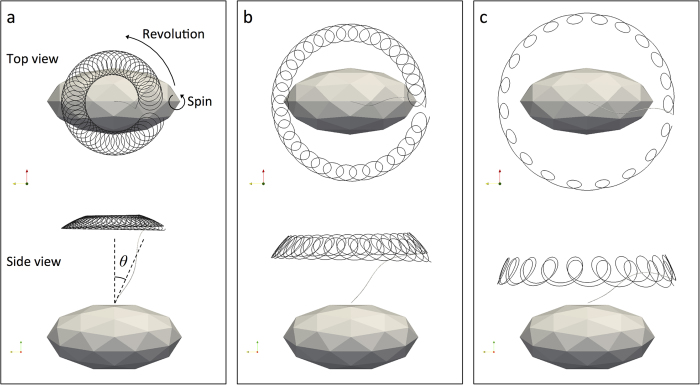
Trajectories of the flagellar tip of the *E. coli* model at selected phases, showing the filament motion consisted of two types of rotation: *spin* and *revolution*, which resulted in precession. The filament moved gradually to a more horizontal direction (tilt angle *θ* = *π*/2 rad) with time (from a to c). The time interval between (**a**) and (**b**) was ~0.2 sec, and ~0.1 sec between (**b**) and (**c**), by assuming a motor torque of *T*_m_ = 10^3^ pN∙nm and fluid viscosity of *μ* = 10^−3^ Pa∙s. The time scale of the tilt angle change was much longer than that of one spin rotation and one revolution rotation.

**Figure 3:**
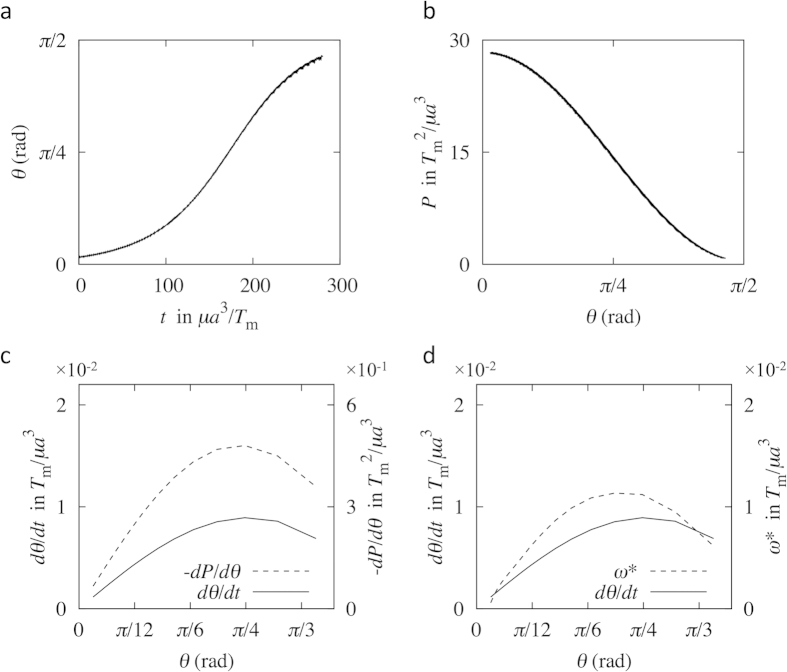
Flagellar behavior in the torque-induced precession of the *E. coli* model. (**a**) Change in the filament tilt angle *θ* over time. (**b**) Change in the motor power *P* over the tilt angle. (**c**) Comparison of the gradient –d*P*/d*θ* and angular velocity *dθ*/*dt*. (**d**) Comparison of the velocity gradient ω^*^ and angular velocity *dθ*/*dt*.

First, we performed a full numerical simulation of the filament motion using the boundary element method (BEM) (see Materials and Methods) to investigate the torque-induced precession of the filament. Figure 2 shows sample trajectories of the flagellar tip of the *E. coli* model during one revolution at selected phases. Both the revolution and spin of the filament define the precession. During precession, the filament moved gradually to a more horizontal direction (*θ* = *π*/2 rad). The time interval between Fig. 2a,b was ~0.2 sec, and ~0.1 sec between Fig. 2b,c, assuming a motor torque of *T*_m_ = 10^3^ pN∙nm and fluid viscosity of *μ* = 10^−3^ Pa∙s. Moreover, the time interval between the tilt angles *θ* = 3° and 85° was ~1 sec. Since the time needed for one spin rotation and one revolution rotation in Fig. 2a were ~0.001 sec and ~0.04 sec, respectively, the time scale of the tilt angle change was much longer than that of one spin rotation and one revolution rotation. We investigated this relatively slow change in the tilt angle over time in detail for the *E. coli* model, in which the initial tilt angle was set to be a non-zero value (*θ* = 0.05 rad) to break the axisymmetry; the results are shown in Fig. 3a. The tilt angle increased over time, exhibiting a maximum gradient at approximately *θ* = *π*/4 rad.

In an attempt to understand the basic mechanism of the slow change in the tilt angle, we first calculated the power *P* generated by the motor, which is equivalent to the power dissipated by the fluid viscosity. The results are shown in Fig. 3b; the calculated power decreased over the tilt angle monotonically. To compare the rate of change in *θ* and *P*, Fig. 3c shows the two gradients, d*θ*/d*t* and −d*P*/d*θ*, over the tilt angle. The two curves show similar tendencies, having a maximum gradient at approximately *θ* = *π*/4 rad. These results indicates that, during the slow change in the tilt angle *θ*, the filament moved so as to minimize the power *P*, and the rate of change in *θ* was associated with that in *P*. If the flagellar motor rotates in the opposite direction, however, the filament moves back to *θ* = 0 due to the time-reversibility of Stokes flow. In this situation, the power *P* increases during the filament movement. Thus, the slow change in the tilt angle over time cannot be explained by the minimization of the power *P*.

As an alternative way to explain the slow change in the tilt angle, we analyzed the flow field generated by the hydrodynamic interaction between the rotating flagellum and the wall. Here, we simplified the problem settings and compared the angular velocities between the simplified system and the full simulation. First, the thrust force of the flagellum, calculated by integrating the traction force over the filament surface, was modeled as a point force at the flagellum center. The velocity field generated by the hydrodynamic interaction between the point force and the wall was calculated using the half-space kernel **G**, which is the Green’s function for a flow bounded by an infinite plane wall^14^. Last, we extracted the velocity component normal with respect to the filament axis, and calculated the velocity gradient ω^*^ by dividing the normal velocity by the distance between the filament junction and the point force. Figure 3d shows a comparison between the results obtained for ω^*^ and the actual angular velocity *dθ*/*dt*. The ω^*^ curve shows a similar tendency as that of *dθ*/*dt*. If the flagellar motor rotates in the opposite direction, in this case, the point force and the flow field are generated in the opposite direction. Thus, the filament moves back to *θ* = 0 by the opposite flow field, which is consistent with the time-reversibility of Stokes flow. These results illustrate that the slow change in the tilt angle of the flagellum over time can be well explained by the hydrodynamic interaction between the rotating flagellum and the wall. The results also indicate that the angular velocity increases with the magnitude of the thrust force.

The bending rigidity due to the hook and the flagellum determines the final tilt angle by the balance of the bending torque, and the hydrodynamic torque generated by the interaction between the rotating flagellum and the wall. In this work, we neglected the bending rigidity of the hook reported in a former study^15^ for simplicity, because the bending torque is believed theoretically not to affect the spin and revolution motion significantly under given tilt angle condition, due to the orthogonality between the bending torque axis and the rotation axes of spin and revolution.

The reason why the precession motion occurs can be explained qualitatively as follows. When the tilt angle is zero, the direction of the filament axis is parallel to the direction of the applied torque, and the filament exhibits only the spin motion with the maximum angular velocity. In many real cases, however, the filament tilt angle becomes non-zero due to the hydrodynamic torque generated by the interaction between the rotating flagellum and the wall. When the tilt angle is non-zero, the flagellar hook acts as a torque diverter, like a universal joint. A part of the applied torque is transmitted to the direction of the filament axis, which induces the spin angular velocity. The remaining torque induces the revolution angular velocity. These two types of rotation result in the precession. The ratio of the revolution angular velocity to the spin angular velocity depends on the tilt angle and filament geometry, but not on the applied torque, which will be discussed in detail later.

**Figure 4:**
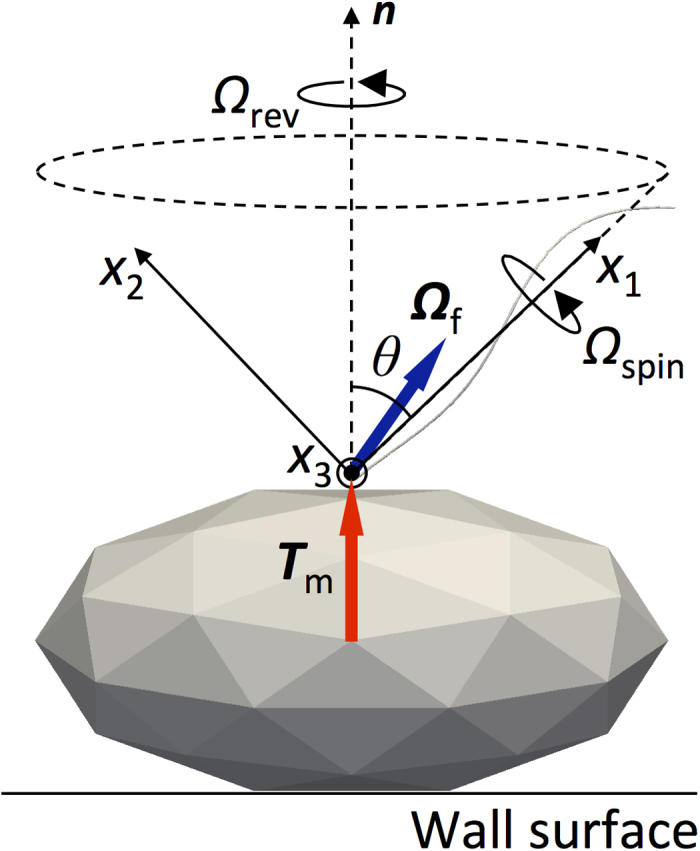
Definition of spin angular velocity *Ω*_spin_ and revolution angular velocity *Ω*_rev_.

Next, we examined the angular velocity of revolution and spin during the precession. The coordinate system defined in Fig. 4 was used to describe the relationship between revolution and spin. In this system, the origin is at the junction, and the *x*_1_ axis is in the same direction as the principal axis of the filament. The *x*_2_ axis is perpendicular to the *x*_1_ axis and in a plane that contains both the *x*_1_ axis and the normal vector ***n*** at the junction. The angular velocity of flagellar filament, ***Ω***_f_, has three components (*Ω*_1_, *Ω*_2_, *Ω*_3_), where *x*_3_ is perpendicular with respect to *x*_1_ and *x*_2_.

To define the spin angular velocity ***Ω***_spin_ and the revolution angular velocity ***Ω***_rev_, we first consider two vectors ***Ω***_1_ and ***Ω***_2_, projection vectors of ***Ω***_f_ onto the *x*_1_ and *x*_2_ axis, respectively; ***Ω***_1_ = *Ω*_1_***e***_1_ = (***Ω***_f_ ∙ ***e***_1_)***e***_1_ and ***Ω***_2_ = *Ω*_2_***e***_2_ = (***Ω***_f_ ∙ ***e***_2_)***e***_2_, where ***e***_1_ and ***e***_2_ are the base vectors. Because the magnitude of the projection vector of ***Ω***_f_ onto the *x*_3_ axis is much smaller than *Ω*_1_ and *Ω*_2_, we approximate***Ω***_f_ as ***Ω***_f_ ≈ ***Ω***_1_ + ***Ω***_2_. We define the spin of the filament as its rotary motion about the x_1_ axis, i.e. ***Ω***_spin_ = ***Ω***_1_. In contrast, the revolution of the filament is often defined by its rotary motion about the ***n*** axis, i.e. ***Ω***_rev_ = *Ω*_rev_***n***. Due to geometric constraints, the revolution angular velocity is given as *Ω*_rev_ = *Ω*_2_/sin*θ*.

**Figure 5:**
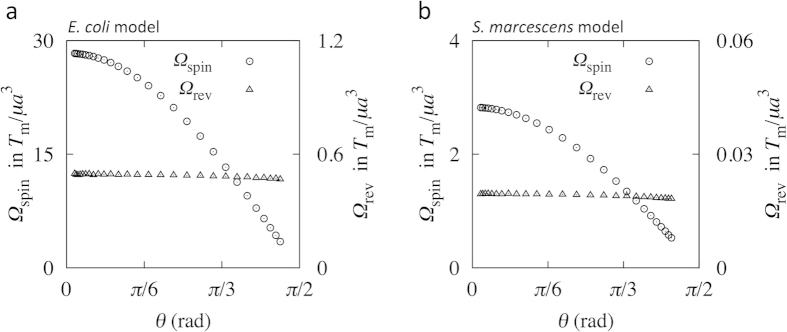
Angular velocities over tilt angle *θ*, obtained using full boundary element method (BEM) simulations for (**a**) the *E*. *coli* model and for (**b**) the *S. marcescens* model.

**Figure 6:**
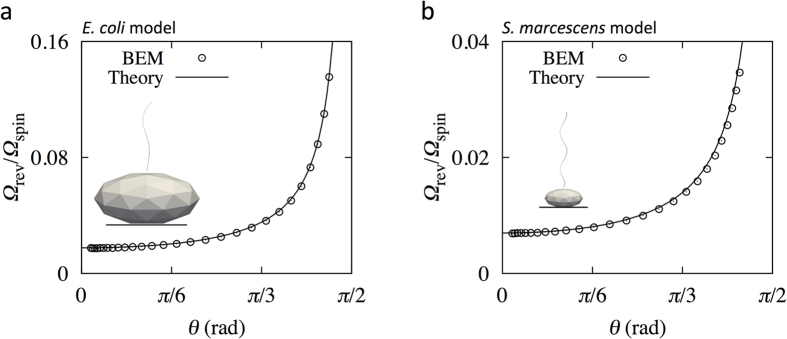
Comparison of *Ω*_rev_/*Ω*_spin_ between the full BEM simulation and theory for (**a**) the *E*. *coli* model and for (**b**) the *S. marcescens* model.

We first show the results from the full BEM simulation. From the angular velocity ***Ω***_f_ obtained by the full simulation, we calculated the spin angular velocity *Ω*_spin_ and revolution angular velocity *Ω*_rev_ for the *E. coli* model, as shown in Fig. 5a; the time-averaged values are shown in intervals of ∆*t* = 10, to smooth high-frequency noise, which corresponds to ~0.03 sec in actual time under the conditions of a motor torque *T*_m_ = 10^3^ pN∙nm and fluid viscosity *μ* = 10^−3^ Pa∙s. The spin angular velocity decreased with increasing tilt angle *θ*, whereas the revolution angular velocity remained nearly constant, regardless of the tilt angle. The revolution angular velocity was markedly smaller than the spin angular velocity. Similar trends were also observed for the *S. marcescens* model, as shown in Fig. 5b, where the time-averaged values are shown in intervals of ∆*t* = 100 (~0.3 sec). The circles in Fig. 6a,b indicate the angular velocity ratio *Ω*_rev_/*Ω*_spin_ as a function of the tilt angle for the *E. coli* and *S. marcescens* models, respectively. A gradual change in the angular velocity ratio was observed over the low tilt angle range, whereas a rapid increase was observed for the high tilt angle regime.

Next, we seek to find a simple theoretical description of the torque-induced precession of bacterial flagella. In the Stokes flow regime, the applied torque ***T*** = (*T*_1_, *T*_2_, *T*_3_) and the induced angular velocity ***Ω*** = (*Ω*_1_, *Ω*_2_, *Ω*_3_) of a rigidly rotating object, without translational velocity, are linearly related as *T*_i_ = *R*_ij_*Ω*_j_^16^. *R*_ij_ is the rotational resistance tensor of the object, whose elements depend on the object shape and the choice of coordinate system. When we use the coordinate system defined in Fig. 4, *T*_3_ is zero; the nondiagonal elements of matrix *R*_ij_ are much smaller than its diagonal elements, due to the slender filament. Thus, the equation for rotational motion may be simplified as *T*_1_ = *R*_11_*Ω*_1_ and *T*_2_ = *R*_22_*Ω*_2_. Using the relationship *T*_1_ = *T*_m_ cos*θ* and *T*_2_ = *T*_m_ sin*θ*, we obtain *Ω*_1_ = *T*_m_ cos*θ*/*R*_11_ and *Ω*_2_ = *T*_m_ sin*θ*/*R*_22_. Using *Ω*_spin_ = *Ω*_1_ and *Ω*_rev_ = *Ω*_2_/sin*θ*, we eventually obtain


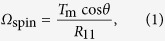



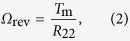



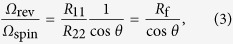


where *R*_f_ = *R*_11_/*R*_22_ is a constant determined by the filament shape. From Eq. (2), the revolution angular velocity *Ω*_rev_ does not depend on the tilt angle *θ*, which is consistent with the results shown in Fig. 5a,b. Equation (3) also indicates that the angular velocity ratio *Ω*_rev_/*Ω*_spin_ does not depend on the applied motor torque *T*_m_.

To verify the derived theoretical predictions, we compare the angular velocity ratio *Ω*_rev_/*Ω*_spin_ of Eq. (3) with those obtained using the full BEM simulation. In Eq. (3), we calculated the resistance coefficients *R*_11_ and *R*_22_ from the BEM numerical simulation. We note that *R*_11_ has a unique value, whereas *R*_22_ does not, due to the axial asymmetry of the filaments. Thus, we averaged *R*_22_ for all angle orientations. The *R*_f_ value was 0.0177 for the *E. coli* model and 0.00698 for the *S. marcescens* model. Figure 6a,b show a comparison of the angular velocity ratio *Ω*_rev_/*Ω*_spin_ between BEM simulations and theory for the *E. coli* and *S. marcescens* models, respectively. The theoretical and simulation results were in agreement for both models, which demonstrates that the proposed theory provides an accurate description of the behavior of torque-induced flagellar precession of different types of bacteria.

To demonstrate the usefulness of our theory, we compared it with our experimental results. In the experiments, we measured the spin angular velocity *Ω*_spin_, revolution angular velocity *Ω*_rev_, revolution radius *r*_b_, and the height of an attached gold nanoparticle from the cell surface, using the stuck cell and bead assay techniques (see Materials and Methods).

**Figure 7:**
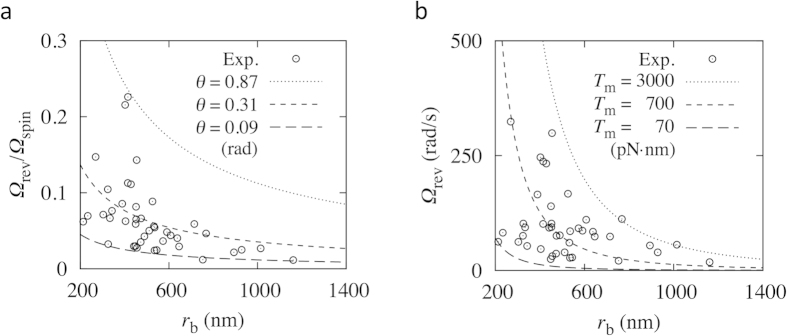
Comparison of the theoretical and experimental results of a chimeric motor expressed in *E. coli*. (**a**) Angular velocity ratio *Ω*_rev_/*Ω*_spin_ over revolution radius *r*_b_. (**b**) Revolution angular velocity *Ω*_rev_ over revolution radius *r*_b_, with a tilt angle *θ* = 0.31 rad (18°) and fluid viscosity *μ* = 9.6 × 10^−4^ Pa∙s.

The angular velocity experimental results of *E. coli.* (n = 42) for various revolution radii *r*_b_ are plotted in Fig. 7a,b; the precession of the gold nanoparticle was in a steady state with respect to the tilting movement (*θ* direction), retaining a nearly constant tilt angle for each cell experiment. Both *Ω*_rev_ and *Ω*_rev_/*Ω*_spin_ decreased with increasing *r*_b_. In comparing the theoretical and experimental results, we need another assumption about the attachment point of the gold nanoparticle. Based on the experimental results of the distance between the base of a filament and the attachment point of the gold nanoparticle along the filament, we assumed that the gold nanoparticles adhered around the tip of the filament, with each filament length approximated as *L* = *r*_b_/sin*θ*. The other parameters were as those listed for the *E*. *coli* model (Fig. 1b). Using these parameters and the BEM, we calculated the resistance coefficient of the filament with an attached 250 nm-diameter gold nanoparticle as a function of *r*_b_ and *θ*. Figure 7a shows the theoretical results for Eq. (3) for three tilt angles; *Ω*_rev_/*Ω*_spin_ decreased with increasing *r*_b_, similar to the experimental results. From Fig. 7a, the filament tilt angle was estimated to be approximately 0.31 rad (18°). We note that we also estimated *θ* directly by using experimental results of *r*_b_ and the height of the gold nanoparticle from the cell surface, to compare *θ* between the experiment and the theory. The tilt angle *θ* was estimated to have the average value of 0.21 rad (12.1°) with the standard deviation of 0.06 rad (±3.3°), which is not significantly different from the theoretical result.

Motor torque can also be estimated by comparing the theoretical and experimental results. The curves in Fig. 7b show the results obtained using Eq. (2), with *θ* = 0.31 rad (18°) for different motor torque conditions; *Ω*_rev_ decreased as *r*_b_ increased, in agreement with the experimental results. Based on these results, the motor torque was estimated to be ~700 pN∙nm.

In summary, we provided a physical description of the torque-induced precession of bacterial flagella by combining full BEM simulations, theoretical modeling, and experiments. Our simple theory agreed well with the full BEM simulations, allowing the filament tilt angle and applied motor torque to be predicted, based on experimental flagellar precession data. The knowledge obtained is important in understanding mechanical properties of the bacterial motor and hook.

## Materials and Methods

### Bacterial model

In this study, the bacterium shape is modeled as a prolate spheroidal cell body of polar radius *a* and equatorial radius *b*, with a single lateral flagellar filament. The filament has a helical centerline with a circular cross-section of radius *c*. The centerline is described by the helix model of Higdon^17^ as follows:


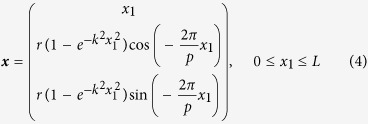


The model defines helix length *L*, helix maximum radius *r*, pitch *p*, and a constant *k* that determines how quickly the helix grows to its maximum radius with increasing distance from the junction. Figure 1b shows the *E*. *coli* and *S. marcescens* bacterial models; the parameters, derived from the literatures^18^,^19^,^20^, are also shown in the figure. Since the data of filament radius *c* of *S. marcescens* was not available, it was set to the same value as the *E. coli* model. The helix length *L* was set to 2 *μ*m for both models in the present study.

### Basic equations in numerical simulation

The flow field around the filament was assumed to be Stokes flow, due to its small size. As such, the velocity field ***u***(***x***) in a fluid with viscosity *μ* can be described by the boundary integral equation^21^:


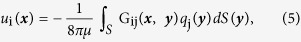


where *S* is the surface of the bacterium, ***q*** is the traction force, and **G** is the Green’s function for a flow bounded by an infinite plane wall^14^. The velocity boundary conditions can be written as


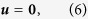


for the stuck cell body surface, and


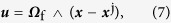


for the filament surface, where *x*^j^ is the position of the junction.

The angular velocity of the rigidly rotating filament, ***Ω***_f_, is determined so as to satisfy the following torque balance between the applied motor torque ***T***_m_ and the drag torque due to fluid viscosity:


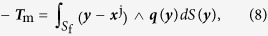


where *S*_f_ is the surface of the filament. We neglected the effect of Brownian motion, because the displacement induced by the motor torque is much larger than the Brownian displacement.

### Numerical methods

Similar to the work of Ishikawa *et al.*^22^, we used the boundary element method (BEM) to discretize the equations. The discretization of Eq. (5), the velocity boundary conditions of Eqs. (6) and (7) with respect to the bacterial surface, and the torque balance of Eq. (8) together yield a system of linear equations that can be solved numerically. A computational mesh was generated for the bacterial surface, in which the cell body and filament surfaces were composed of 80 and 510 triangular elements, respectively. The integration in Eq. (5) was performed on a triangular element using a 28-point Gaussian polynomial, and the singularity in the integration was solved analytically^21^. Time-elapsed filament motion was determined using the fourth-order Adams–Bashforth method. All physical quantities were nondimensionalized using a cell body polar radius *a*, fluid viscosity *μ*, and applied motor torque magnitude *T*_m_.

### Bacterial strains and experimental procedure

The experimental procedure used was similar to that used in our former study^5^; thus, we provide a brief explanation here. Chimeric motors were expressed in *E. coli* strain JHC36 (Δ*cheY*, *fliC*^*sticky*^, Δ*pilA*, Δ*motAmotB*) with a plasmid pTH200 (PomA, PotB), inducible by IPTG, as described in a previous study^5^. Cells were grown from frozen stock, with shaking in T-broth (1% Bacto^TM^ tryptone, 0.5% NaCl) containing 25 *μ*g/mL chloramphenicol and 0.05 mM IPTG at 31 °C for 5 h. Rotation of the truncated flagella was examined using bead assays, as described previously^5^, with some modifications. Gold nanoparticles (diameter: 250 nm; BBI Solutions, Cardiff, UK) were attached to truncated flagella; the particle positions were observed using an inverted microscope (IX70, Olympus, Tokyo, Japan) and recorded using a high-speed camera (HAS-220, Ditect, Tokyo, Japan) at a sampling rate of 2000 frames per second. Experiments were performed at room temperature, 23 °C. The spin angular velocity *Ω*_spin_, revolution angular velocity *Ω*_rev_, and revolution radius *r*_b_ were obtained from the gold nanoparticle position data set for each cell (see Supplementary Table S1 online).

## Additional Information

**How to cite this article**: Shimogonya, Y. *et al.* Torque-induced precession of bacterial flagella. *Sci. Rep.* **5**, 18488; doi: 10.1038/srep18488 (2015).

## Supplementary Material

Supplementary Video S1

Supplementary Video S2

Supplementary Video S3

Supplementary Information
