# Supplementary material for: Torque-induced precession of bacterial flagella
Source: Sci Rep. 2015 Dec 22;5:18488. doi: 10.1038/srep18488 (PMC4686982; doi:10.1038/srep18488)
Supplement: Supplementary Information [file srep18488-s4.pdf]

Supplementary Information for

Torque-induced precession of bacterial flagella

Yuji Shimogonya[1*], Yoichiro Sawano[2], Hiromichi Wakebe[2], Yuichi Inoue[3], Akihiko Ishijima[3,4] & Takuji Ishikawa[5]

[1]Frontier Research Institute for Interdisciplinary Sciences, Tohoku University, Sendai 980-8578, Japan. [2]Graduate School of Life Sciences, Tohoku University, Sendai 980-8577, Japan. [3]Institute of Multidisciplinary Research for Advanced Materials, Tohoku University, Sendai 980-8577, Japan. [4]Graduate School of Frontier Biosciences, Osaka University, Osaka 565-0871, Japan. [5]Department of Bioengineering and Robotics, Tohoku University, Sendai 980-8579, Japan.

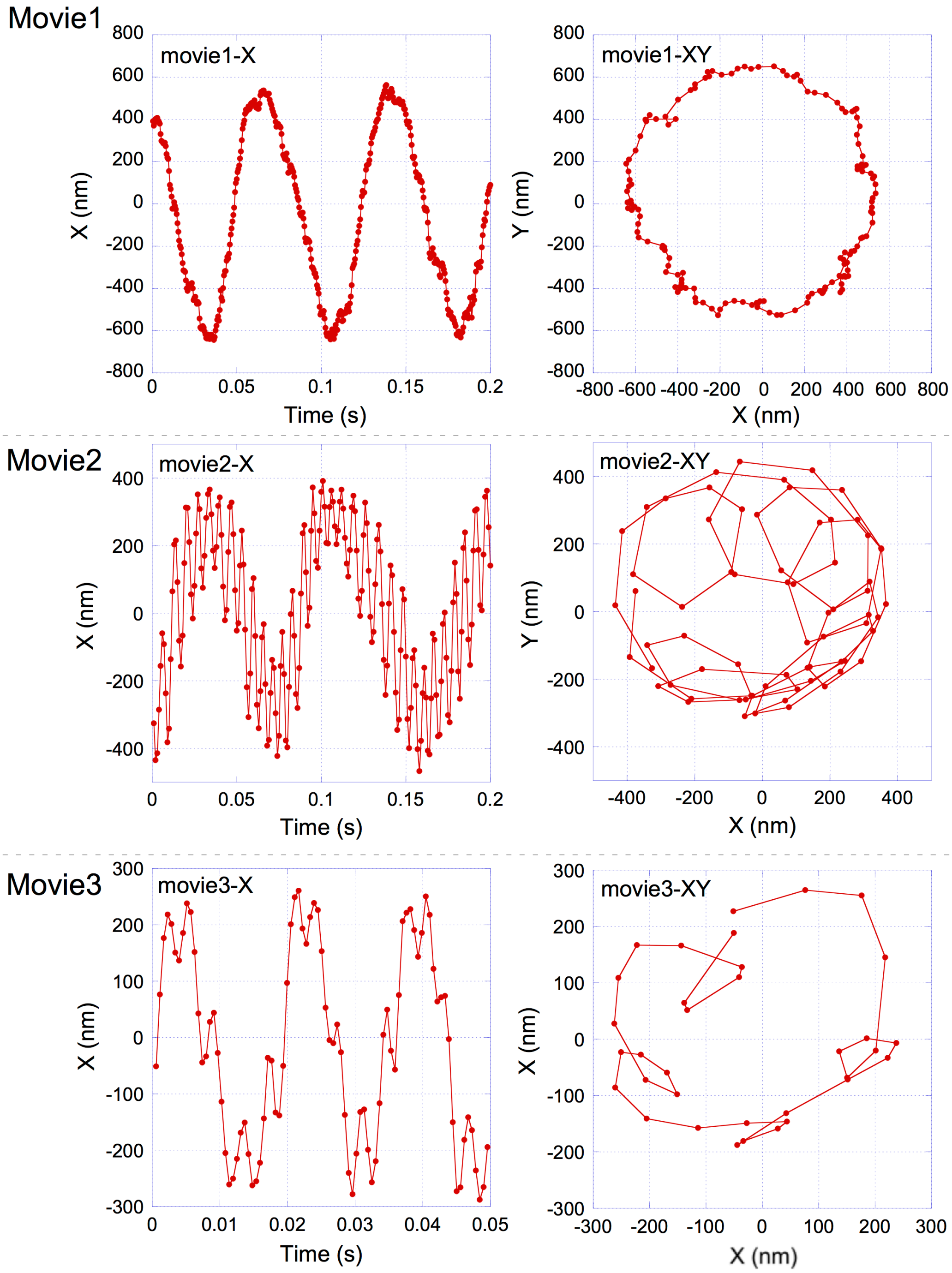

Figure S1. Traces of the videos S1-S3. Centroids of the gold nanoparticles in the videos S1-S3 were analyzed. Time courses of the X-position (Left) and XY trajectories in the first revolution (Right) were shown in video S1 (upper row), video S2 (middle row), video S3 (bottom row). Revolution speeds for videos S1-S3 were ~13 Hz, ~11 Hz, and ~57 Hz, respectively. Spin speed and spin radius were ~170 Hz and ~17 nm for video S1, ~150 Hz and ~82 nm for video S2, ~280 Hz and ~50 nm for video S3.

Table S1. Experimental data of the flagellar precession of *E. coli*. The spin angular velocity Ω_{spin}, revolution angular velocity Ω_{rev}, and revolution radius r_b were obtained with a probe of the 250 nm-diameter gold nanoparticle for each cell.

Case	Revolution radius r_b (nm)	Spin angular velocity Ω_{spin} (rad/s)	Revolution angular velocity Ω_{rev} (rad/s)
1	526	1885	166.8
2	573	2513	91.7
3	414	899	101.3
4	591	1797	86.7
5	455	2092	299.2
6	475	1144	75.7
7	415	1049	237.1
8	402	1144	246.4
9	638	2092	84.3
10	453	1571	102.2
11	269	2205	324.2
12	1161	1571	18.1
13	929	1571	39.3
14	429	2094	232.7
15	714	1257	73.9
16	489	1795	76.6
17	1014	2094	55.9
18	768	2417	112.2
19	450	2371	139.6
20	893	2513	54.6
21	536	1571	85.5
22	607	2513	110.2
23	304	872	62.0
24	213	1010	62.4
25	753	1795	21.3
26	456	1126	31.1
27	532	1079	60.3
28	442	3142	93.0
29	343	702	53.5
30	334	1405	93.6
31	473	1047	36.8
32	545	1148	28.1
33	405	746	46.7
34	646	2513	73.9
35	236	1182	82.3
36	451	1158	94.4
37	326	3142	102.2
38	390	1933	165.3
39	535	1142	27.6
40	449	832	24.8
41	324	723	75.6
42	511	783	39.2
